# The Possibilities of Prosthetic Dentistry

**Published:** 1897-12

**Authors:** E. J. Perry

**Affiliations:** Chicago, Ill.


ARTICLE III.

### THE POSSIBILITIES OF PROSTHETIC DENTISTRY.

BY E. J. PERRY, D. D. L., CHICAGO, ILL.

**Read before the Northern Illinois Dental Society Rockford, October, 1897.**

A consideration of the possibilities of prosthetic dental art might lead one to the discussion of impracticable ideas, but then the question comes back to what extent are ideas impracticable? This society must and is intended to deal with practical things. My friends, the practical in this world and in our chosen field must come nearer to the ideal. There Should not be such a wide interval between ideal and practical excellence, but a perfect and harmonious blending of both qualities. A conscientious study of prosthetic dental art will be found to require, in order to attain the esthetic and the useful without sacrificing one to the other qualities of mind not common to the artisan. A creative quality of mind is indispensable to the construction of a full denture. A person with edentulous jaws is in a sorry plight. He is indeed unfortunate. The features are changed and distored. The brilliancy of the beauty of the face devine is gone. His digestion impaired; his days shortened. To whom shall he go? To the dental mechanic? No, but alas, he has no other place where he can go. Why don't the profession take this unfortunate person's case to heart? Who is to blame that so many persons wear false teeth, and so few wear artificial ones ? Is the person so afflicted or the profession? The profesion is to blame. Let us go back a few years. Let us be reminiscent for a time. We will see, I think, wherein lies the cause. When your essayist began to study dentistry, the metals, gold, silver and platinum, where jnst packing their trunks in disgust and preparing to leave the laboratory. I was introduced in the most formal way, and away they went. Vulcanite had run them out where for long years they had held full sway. My preceptor served four years' apprenticeship in the laboratory alone, I served one year, then went to college. What did I find in the laboratory there? Vulcanite was supreme. Gold and silver and platinum had gone, and no one was left to speak of their good qualities. Their biographies were read, that was all. Where was the unfortunate individual with the edentulous jaws. He was steered up against Mr. Vulcanite. His predecessors in trouble had been taught by the former knights of the laboratory to pay handsomely for services rendered. Hence it was easy to get a big price for vulcanite, and the work quickly done. Then came in the Barbarians and the Philistines, and competition ran down the price. The best men went into the operating room and the breach between the two widened. This was the state of affairs up to the advent of modern crown and bridge work. Now the future looks bright. The metals with their bright faces have forced terms with the despot vulcanite and the operator, however high his dignity, can sometimes be found in the laboratory, and to my optimistic vision the possibilities of prosthetic dental art are indeed ranging into the realm of real probabilities. Much is to be done. Line upon line and precept upon precept are to be written. We must begin at the societies and colleges.

Ability of a high order is coming into the laboratory, the electric furnace, electric lathe, the various methods of making crown and bridges and dentures and the facination of the dental ceramic art are forcing up the standard of prosthetic work. Crown and bridge work have lifted into the free air and pure sunshine the banner of prosthetic dental art. It has washed up the benches and cleaned up the laboratory, so that its dingy walls and disgusting filth are no longer its distinctive features. But instead, the laboratory is neat and clean, artistic and convenient in arrangement and altogether in a first class office the most facinating place in which to work. The metals have done this. Ceramics are now, and are in the future to cut a large figure in dental practice. They exact of its servants neatness and methodical precision. Crown work of any sort exacts of the dentist not alone mechanical excellence, but artistic consep-tion, ingenious and creative skill, esthetic tastes, and the discriminating power of application. The importance of prosthetic dental art to humanity is forcing itself into the heart and mind of the profession. Not the artisan, but the artist, the doctor, the dentist, who combines in one brain case all these in one. He is thinking, and the aforesaid unfortunate person with toothless and expressionless face is being steered up against a very different proposition. Doubtless the almighty dollar has a finger in this pie and is an important factor in the case, but no matter, the results are the same, and the credit is not discounted. I say to you if per chance you are here, you who extract aching or devitalized teeth, you who recommend rubber as the best base for artifical teeth, or you lily fingered operators who never do any plate work, who do not go into the laboratory except to smoke or black your boots, who have laboratories only for the storage of filth, soiled towels, cast-off clothes and broken furniture; I say to you whoever you are or where, look to your laurels the new day has dawned; the triumphal car is coming; an exacting public will disown you, and if you should fall you would be trampled on and you cannot see the procession for dust. Go from this meeting ye who fall under this description and clean up your dark and filthy laboratories; put in neat appliances and modern apparatus; get in line of progress make your own crown and bridges; get hold of porcelain; catch on to ceramics; stop putting in those ready made hand me down crowns; get some works on metallurgy and ceramics; give not the hours after you have done a day's work in the operating room, but of your best time and best thought; begin by urging those who can pay to have continuous gum or gold work and their number will grow.

But you must believe what you say and you must be able to do the work. You cannot hire it done; you can hire an artician, but you will never in this life succeed until you thoroughly master the subject yourself. You cannot even hire it done properly till you can do it yourself. You must be the designer, the creator. You must possess the whole case; you must be the sole master and what you can accomplish will bring to you great joy. The profession must bring this to the great common man. How can it be done? How can the common man be made to appreciate artistic prosthetic dental art? Certainly the first requisite in the matter must be a thorough practical knowledge and appreciation of the artistic in prosthetics on the part of the dentist himself; it must be a conviction with him, it must come from the heart and head both; he must be able to see the beauties and the utilities of his work ahead of his patient; when he says to his toothless patient a continuous gum set is best, he must have a better reason in his mind than he thought he is rich and can pay for it. The most of our patients, or at least a goodly number can pay for continuous gums if they are convinced that it is infinitely better for them in whatever way considered. No dentist who is an artist in prosthetic work would himself wear a rubber plate for a full denture. I know patients who would take in washing if need be to get a continuous gum work. This work must be done by the dentist; if he cant do it or sends to the laboratory man an occasional set for some rich patient, he gets back a continuous set less the art. The case does him little good or possibly the patient either, so the whole matter rests with the dentist. He is timid; he is stingy; he is lazy; he is ignorant; he is incompetent; he lacks enterprise; he is not honest; some of these or all of them he is or the people who look for better things would have them. The profession as a whole is to blame for whatever lack of true art there may be in prosthetic work of to-day. You say the people are looking for a cheap thing. This is not true. They are looking for the best as much as ever. You have nothing but a cheap thing to offer them. The dentist himself is the most important factor in raising the standard of professional excellence in a community. The people will not force up the standard for him, but they will recognize and support him by an intelligent appreciation of every effort he may rightly make to elevate and ennoble his profession.

Merit, art, skill, industry and professional excellence do not go unrewarded. Did you ever think how few sets of teeth you have made and inserted that were just right. and how rarely a good piece of work of any sort went unrewarded? Have you so much conceit as to think you cannot be outdone in prosthetic work, either in its design, or its mechanical perfection? Do you think the standard is already up, or don't you think it will pay you to hoist it? Let us briefly consider a few points of advantage which lie in the way of say gold for partial sets, or continuous gums for full plate. No one who ever wrote on the subject but emphasizes the fact that a plate covering the tissues for the purpose of carrying artificial teeth should be a good conductor of thermal changes. Here, then, rubber is contraindicted. Gold, platinum or porcelain therefore are indicated. The material of the plate should also possess the greatest possible strength in the least possible space. Again, rubber fails to fill the requirements of the case, the metals do not. The material of the plate should also be pure and non-oxidizable. Here rubber fails also and twenty karat gold highly finished and triple plated for the partial set and pure platinum and porcelain for the full fill all the requirements of the case to perfection. In continuous gum work the artist has the largest possible liberty in the arrangement of the teeth with exact reference to the artistic requirements of the case, using art to conceal art. He cannot do this with any possible means with rubber. Perhaps one of the most important points gained in continuous gum is in the gum.

Arrange teeth in continuous gum work as you may, they look infinitely better than the same arrangement in rubber. One might pass, the other be simply hideous. The advantages of a correct occlusion are easily obtained also in continuous gum for the metal plate can be used after the pins are soldered to the plate and a gum colored wax used, and not only an absolutely correct occlusion but a study of the restoration of the lost expression can be made in the mouth in a way not possible under any other circumstances. In partial sets the plain plate teeth can be tipped with gold as are facings in crowns and bridges, thus securing durability and strength, either the adhesion plate or broad clasps can be used and in some cases the Condict fastenings are incomparable. Bridge work has lessened the number of partial sets and often the four incisors can be carried in this way, not on two cuspids however. It is not my purpose to detail any method. The discussion ought to bring out much detail. My purpose is to awaken you to the important fact that the prosthetic half of dentistry is to take its rightful place and the results that are possible you are to assist in realizing.

I want you to realize what the possibilities of prosthetic dental art are. What the forces are that are working out a higher destiny for it. Just turn your studies into this channel.

One more word. In some quarters I hear that teachers encourage the separation of the two great branches, operative and prosthetic dentistry. A further specialization of dentistry! An eminent teacher told me that prosthetic dentistry should not be taught in dental colleges, or at least, no attempt made to finish men for practice. A special school for this work, that the practitioner should do only operative or prosthetic work. I cannot see how any one can so argue. Where we are lame is in the fact that the colleges do not properly fit men to practice prosthetic art, and the practitioner does not spend enough time or study in the laboratory. There is too much specializing. Men do not grow symetrically in practice. They advance as they think in the groove in which they work, and when men get into a groove as the years go by, that groove gets narrower and the man resembles the groove. A further specializing will destroy the profession. The prosthetic man must work in the mouth and when he grows in this work of prosthetic dental art look for a growth in his operative work also.—*Dental Review*.

